# The Swedish dental health register - validation study of remaining and intact teeth

**DOI:** 10.1186/s12903-019-0804-7

**Published:** 2019-06-17

**Authors:** Rickard Ljung, Frida Lundgren, Marianne Appelquist, Andreas Cederlund

**Affiliations:** 1 Unit of Epidemiology, Institute of Environmental Medicine, Karolinska Institutet, PO Box 210, SE-171 77 Stockholm, Sweden; 2 National Board of Health and Welfare, Stockholm, Sweden; 3 Public Dental Health Stockholm county council, Stockholm, Sweden

**Keywords:** Caries, Dental health, Health care utilization, Inequalities in health

## Abstract

**Background:**

Sweden has a long tradition of nationwide registers enabling population-based research of high quality and validity. We aimed to describe the content and validity of reported number of remaining and intact teeth in the Swedish Dental Health Register and report some descriptive data on dental health care utilization.

**Methods:**

The Swedish Dental Health Register was initiated in July 1st 2008 and contains individual data on dental health care to the whole adult population of Sweden. The dental care given freely to children and young adults is not included. Descriptive data on remaining, intact teeth and dental health care utilization is presented by proportion of the population stratified by sex and age. We conducted a validation study, by manual review of randomly sampled 1500 dental health visits records, to assess reported number of teeth to the register with what was actually recorded in the dental health care record (gold standard), analyzed by positive predictive value (PPV) and Bland-Altman plots.

**Results:**

Of the Swedish adult population 2014, 2.6 million (69%) men and 2.9 million (76%) women had at least one visit to a dentist during a two-year period 2013 to 2014. More than half of the population up to age 64 have all remaining teeth (28 teeth or more). Of the 1500 requested dental records 1131 (75%) were received. The positive predictive value for patients reported to the register as having at least 1 tooth up to 31 intact teeth was 91.5% (95% confidence interval 89.0–93.5, 567 manually reviewed to be correct out of 620 reported).

**Conclusions:**

For patients coded as having less than 32 intact teeth but not being edentulous the reported number of remaining and intact teeth is to a very high degree correct. However, the correctness for those coded as edentulous or having 32 remaining intact teeth is low and varies substantially by age.

## Background

Poor oral health as defined by diseases of the oral cavity, foremost periodontitis and caries, and limited number of remaining intact teeth constitutes a major global health burden. The distribution of poor oral health is socially patterned as shown by large socioeconomic disparities in oral health within and between countries. [1, 2] There is limited data on oral health and its determinants in the Swedish population [3], but there are indications of major social disparities. [3–8] A large part of the global population is not covered by dental health care, and in settings with high coverage the utilization varies across socioeconomic groups. Sweden has universal free dental health care services for children and young adults up to the year of their 23rd birthday. For adults, from the year they turn 24, dental health care services are financed by state subsidization and patient out-of-pocket payment and a small part by the county council for some specific group in the population. Dental care is to a large extent (60% in the year 2014) financed by the consumers of dental care. [9–11]

Population-based registers facilitate high quality research provided that registration is complete and valid. There is a need for a national register with high coverage, completeness and validity to facilitate continuous monitoring of the distribution of dental health in the population, evaluation of dental health care treatment, and to enable studies of the association between dental health and other diseases. Sweden has a long tradition of nationwide population registers enabling population-based research of high quality. [12] In the other Nordic countries there has been national or regional registers on dental health foremost for children, including a Swedish register on dental implants and another for dental caries and periodontitis, but for long Sweden has not had a national register covering the adult population. [13–16] However, in 2008 the Swedish Dental Health Register was initiated. The register contains individual data on dental health care reimbursed by the State to the whole adult (23 years and older) population of Sweden. [17]

The data collection is administered by the Swedish Insurance Agency as part of the administration of the reimbursement from the State to dental health care facilities for dental services provided to the population. Information on dental health care is monthly transferred to the National Board of Health and Welfare, which is responsible for keeping all national health data registers. The Swedish Dental Health Register is regulated under a legislation issued by the Swedish government and can among others be used for evaluation and planning of health care services, monitoring public health and for research. Electronic reporting is mandatory for the dental health professions. [18]

We aim to describe the content and the validity of reported number of teeth of the Swedish Dental Health Register and to report some descriptive data on dental health care utilization.

## Method

### Dental health care utilization

In the descriptive analyses we used data from the Swedish Dental Health Register for the mean Swedish population in 2014. [19] Dental health care utilization measured as at least one visit during follow-up was calculated for a 1 year period (2014), a 2 year period (2013–2014) and a 3 year period (2012–2014). Due to some lag in reporting and corrections the register is continuously updated. Hence, later versions are more complete for a specific previous year. Also, at the time of the study Sweden had universal free dental health care services for children and young adults up to the year of their 19th birthday, hence we followed the adult population from age 20 and older. From 2017, upper age limit has been gradually expanded and as of 2019, dental care is free for children and young adults up to the year of their 23rd birthday.

### Content of the Swedish dental health register

The register contains clinical data on number of remaining teeth and number of remaining intact teeth, and diagnoses and dental procedures. However, only diagnoses that are subject to an intervention or treatment during the dental visit are recorded. Classifications of diagnoses and dental procedures are determined by The Dental and Pharmaceutical Benefits Agency of Sweden. [20] The agency also determines the level of reimbursement for these procedures.

The register contains four basic categories of information:

- **Patient data**: sex, age, place of residence, personal identity number, citizenship, country of origin, civil status.
- **Dental care data**: name and address of dental clinic or dental practice.
- **Administrative data**: dates of treatment,
- **Clinical data:** diagnoses, tooth number and position, type of procedure, number of remaining teeth, number of intact teeth.

### Validation of the quality of reported number of teeth

We conducted a validation study to compare data reported to the register with what was actually recorded in the dental health care record (gold standard). A total of 1500 dental health visits in the first 2 months of 2014 where selected by stratified random sampling from the Dental Health Register. A letter was sent to each clinic requesting a copy of the dental health care records for the sampled visits. Dental health care records were retrieved from both public and private dental clinics by post, either on paper copies or electronic files, and reviewed by a senior dental hygienist (MA) and a senior dentist (AC). The sampling was done in three different subsets; First, a random sample of 800 visits among those with reported at least 1 tooth up to 31 intact teeth, of these 620 were received (78%), second a random stratified sample by age of 350 records of visits among patients reported with no teeth (edentulous) (received *N* = 225, 64%), and a random stratified sample by age of 350 records of visits among patient with reported all remaining and intact teeth (32 teeth) (received *N* = 286, 82%). Overall response rate was 75% (1131 records received of 1500 sampled).

### Statistical analyses

Data on utilization of dental health care from January 1st, 2012 to December 31st 2014 was presented as proportions stratified by sex and age for the whole Swedish population in 2014. Positive predictive values with 95% confidence intervals, Bland-Altman plot, linear plot and Cohen’s kappa coefficient with 95% confidence intervals were used to present the results of the validation study. In the analyses of Cohen’s kappa coefficient pseudo-observations were added to fill non-square tables with unused categories given a very small weight.

## Results

### Visit to dental services

Of the Swedish adult population 2014, 2,560,000 (69%) men and 2,860,000 (76%) women had at least one visit to a dentist during a two-year period 2013 to 2014 (Table 1). In all age groups, except for those 75 years and older, women had a higher attendance to dental services than men. Around two thirds of young adults aged 20–24 had at least one visit in the last 3 years. The youngest and the very old had the lowest attendance to dental services. The highest attendance was among those aged 65–74 years.

**Table 1:** Proportion of the Swedish mean population in 2014, by sex and age, with at least one record in the Dental Health Register during a 1 year period (2014), a 1 year period (2013–2014) and a 3 year period (2012–2014)

		Proportion of the Swedish population 2014 with at least one record in the Dental Health Register					
		In 2014		In 2014 or 2013		In 2014, 2013 or 2012	
	Population	N	%	N	%	N	%
Women							
20–24	327,449	150,177	45.9	206,211	63.0	222,314	67.9
25–34	609,532	278,785	45.7	400,398	65.7	450,367	73.9
35–44	616,173	334,433	54.3	447,810	72.7	488,905	79.3
45–54	633,789	397,260	62.7	497,411	78.5	530,721	83.7
55–64	571,302	411,739	72.1	477,309	83.5	497,770	87.1
65–74	541,010	422,405	78.1	473,032	87.4	488,600	90.3
75–84	317,377	221,146	69.7	255,062	80.4	268,135	84.5
85+	168,564	77,277	45.8	100,633	59.7	114,817	68.1
Total	3,785,194	2,293,222	60.6	2,857,866	75.5	3,061,629	80.9
Men							
20–24	344,286	140,901	40.9	197,568	57.4	215,563	62.6
25–34	638,657	241,883	37.9	352,615	55.2	402,720	63.1
35–44	636,124	296,878	46.7	401,204	63.1	443,257	69.7
45–54	653,457	363,695	55.7	461,565	70.6	497,934	76.2
55–64	573,299	378,154	66.0	443,625	77.4	466,679	81.4
65–74	524,281	386,447	73.7	435,593	83.1	452,753	86.4
75–84	254,122	177,214	69.7	205,356	80.8	216,349	85.1
85+	87,193	47,280	54.2	61,433	70.5	69,120	79.3
Total	3,711,418	2,032,452	54.8	2,558,959	68.9	2,764,375	74.5

### Edentulous and remaining teeth

More than half of the population up to age 64 had all remaining teeth (28+) (Table 2). Less than 10% of those aged 85 and older had all remaining teeth and around 3 had no teeth left. Among those younger than 35 years around 10% had 20 intact teeth or less.

**Table 2:** Number of remaining teeth and intact teeth of the Swedish population 2014 by sex and age, presented as the number of teeth at the cutoff in the lowest decile (P10), median and highest decile (P90)

					Number remaining teeth				Number intact teeth		
	Population 2014	Patients with reported teeth status in 2014	Edentulous (%)	All remaining (%)	P10	Median	P90	All intact (%)	P10	Median	P90
Women											
20–24	326,946	148,027	341 (0.23)	26,724 (18.1)	27	28	32	9970 (6.7)	21	27	31
25–34	617,535	281,317	976 (0.34)	78,122 (27.8)	27	30	32	21,628 (7.7)	18	26	31
35–44	616,808	308,079	1003 (0.33)	64,519 (20.9)	26	29	32	12,249 (4.0)	14	22	29
45–54	636,427	351,776	1350 (0.38)	44,481 (12.6)	25	28	32	8229 (2.3)	9	17	26
55–64	569,342	346,538	1907 (0.55)	22,546 (6.5)	22	28	31	5806 (1.7)	4	11	21
65–74	547,625	348,819	3575 (1.03)	12,220 (3.5)	17	26	29	4850 (1.4)	0	7	15
75–84	319,868	183,328	4013 (2.19)	4334 (2.4)	10	23	28	2364 (1.3)	0	5	12
85+	168,707	63,581	2144 (3.37)	1837 (2.9)	6	20	27	1073 (1.7)	0	4	11
Total	3,803,258	2,031,465	15,309 (0.75)	254,783 (12.5)	21	28	32	66,169 (3.3)	3	16	28
Men											
20–24	344,583	138,097	248 (0.18)	30,158 (21.8)	28	29	32	9537 (6.9)	21	27	31
25–34	647,598	245,128	785 (0.32)	77,694 (31.7)	28	30	32	19,376 (7.9)	19	26	31
35–44	637,674	271,750	914 (0.34)	68,013 (25.0)	27	30	32	11,464 (4.2)	14	22	30
45–54	656,361	319,785	1248 (0.39)	51,167 (16.0)	26	29	32	7481 (2.3)	10	18	27
55–64	571,928	317,030	1785 (0.56)	27,297 (8.6)	23	28	31	5228 (1.7)	4	12	21
65–74	530,770	317,950	3284 (1.03)	13,399 (4.2)	17	26	30	4224 (1.3)	1	8	17
75–84	258,137	146,409	3225 (2.20)	3939 (2.7)	10	23	28	1898 (1.3)	0	5	14
85+	87,777	38,616	1236 (3.20)	1086 (2.8)	7	20	27	618 (1.6)	0	4	13
Total	3,734,828	1,794,765	12,725 (0.71)	272,753 (15.2)	21	28	32	59,826 (3.3)	3	17	29

### Validation of reported remaining and intact teeth

In total, 98 (43.6, 95% CI 37.0–50.3) out of 225 retrieved dental health records coded as edentulous in the register were assessed as having no teeth based on the dental record. The coding was much better with increasing age; 85% (73.4–92.9) of those 75 years and older coded as edentulous were assessed as correctly edentulous. Of the 286 patients coded as having 32 intact teeth in the register 69 (24.1, 95% CI 19.3–29.5) were assessed as having 32 teeth based on the dental record. For 567 of the 620 records of patients coded as not being edentulous and having less than 32 remaining intact teeth the number of remaining teeth reported to the register was the same as the number of teeth assessed from the validation based on information in the dental record. This corresponds to a positive predictive value of 91.5% (89.0–93.5) for remaining teeth. For 544 of the 620 records of patients coded as not being edentulous and having less than 32 remaining intact teeth the number of intact teeth reported to the register was the same as the number of intact teeth assessed from the validation based on information in the dental record. This corresponds to a positive predictive value of 87.7% (84.9–90.2) for intact teeth. (Table 3).

**Table 3:** Validation of number of remaining intact teeth as recorded in the Dental Health Register by assessment of retrieved dental health records, by number of remaining intact teeth reported to the Dental Health Register in 2014

				Agreement between dental health records and the register					
Remaining intact teeth according to register	Records sampled	Records recieved		Correct	(positive predictive value) % (95% CI)	Incorrect	%	Cannot be assessed	%
0	350	225		98	43.6 (37.0–50.3)	124	(55)	3	[1]
32	350	286	intact	69	24.1 (19.3–29.5)	190	(66)	27	[9]
0 < 32	800	620	remaining	567	91.5 (89.0–93.5)	38	[6]	15	[2]
			intact	544	87.7 (84.9–90.2)	59	[9]	17	[3]

**Fig. 1:**
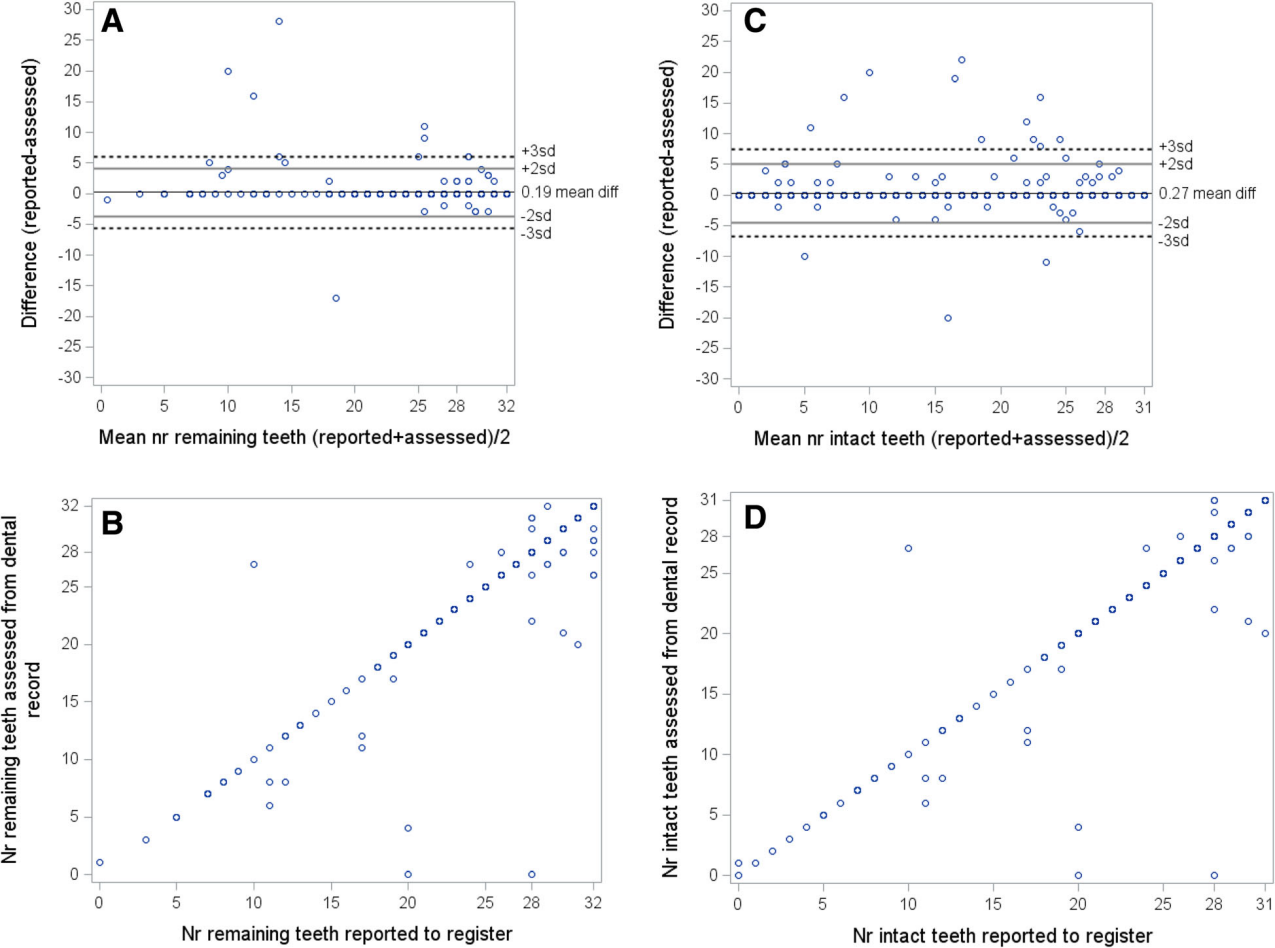
Bland-Altman plots and linear plots of number of reported teeth and number of teeth assessed by manual review of dental health records (599 teeth assessed for comparison of remaining teeth and 595 teeth assessed for comparison of intact teeth). (1A) Bland-Altman plot of remaining teeth, (1B) linear plot of remaining teeth, (1C) Bland-Altman plot of intact teeth, (1D), linear plot intact teeth

A Bland-Altman plot and a linear plot depict the agreement between registered and recorded number of teeth for remaining and intact teeth respectively (Fig. 1). The plots are based on records where numbers of teeth were possible to assess (599 for remaining teeth and 595 for intact teeth), hence for 21 out of the 620 records there was no information on remaining teeth and for 25 out of the 620 there was no information on intact teeth. For remaining teeth the simple kappa was 0.942 (95% CI 0.923–0.962), and the weighted kappa 0.939 (0.910–0.969), for intact teeth the kappa was 0.913 (0.890–0.936) and 0.949 (0.930–0.967) respectively.

## Discussion

The Swedish Dental Health Register provides near to complete national data on dental health care to the adult Swedish population. The positive predictive value of reported teeth is high among those with at least 1 tooth up to 31 intact teeth reported to the register. However, we have pinpointed two major areas where the quality of the register has to be improved considerably; recording of no teeth and recording of all remaining and intact teeth (32 intact teeth). In a random sample the positive predictive value was less than 50% in these groups.

## Strength and limitations

The high quality and validity of the Dental Health Register together with the complete nationwide coverage is a unique source for dental health care services research. This also provides our study with high precision and considerable power in the estimates of the dental health and dental health care utilization of the Swedish population. The four largest computerized reporting systems used in dental health care services in Sweden have predefined default values of either no teeth or 32 teeth. Hence, we hypothesize that some dentists and dental hygienists by routine just click the default value (0 or 32) instead of recording the actual correct number of remaining teeth. This is most probably the explanation of the low validity of 0 and 32 reported teeth. A possible improvement could be to have no default values and thus instead force the recorder to manually fill in the correct number of teeth. We have only validated the recorded number of teeth for that particular sampled dental visit. However, also taking previously recorded values of number of teeth into account would generate a higher concordance between recorded number of teeth and the correct number of teeth assessed by manual review. This indicates that there is no systematic misclassification of number of teeth due to patient or dentist or dental hygienist characteristics. The misclassification more seems to be non-differential. The overall response rate of the validation study was high (75%). However, we cannot totally rule out differential response rate between dentists and dental hygienists recording correctly and those recording less correctly which might have overestimated the quality of the reported number of remaining teeth. The clinical data reported to the register is number of remaining teeth, intact teeth, diagnoses and procedures. However, we have only validated remaining teeth and intact teeth. We argue that remaining intact teeth is a simple and very useful measure of oral health (if the data is correct) to use for dental health surveillance and thus the most important data to validate. However, in the future we hope that reported specific diagnoses and procedures will be validated.

The reform of dental care that was introduced in 2008 included increased governmental subsidies. [9–11] For implementation of the dental reform, the Dental Health Register was introduced, making it necessary for the dentists to register their patients in a computerized system in order to get the subsidies paid out by the governmental agency in charge of the register. However, as the out-of-pocket payment can be substantial, patients with financial distress may opt not to seek dental health care when needed. We have no means to in a systematic way assess the oral health of those not registered in the register.

Until now the sources used for dental health research in Sweden have mainly been regional or local registers or specifically collected data in smaller studies; dental health in the population, [21–23] utilization of dental health care services [24], socioeconomic differences in utilization of health care services, [3–8, 25], risk factors for caries [26], dental health as a risk factor for other disease [27–29]. The Dental Health Register, with data on utilization by age and sex and possible to link to socioeconomic factors is a valuable and very powerful source for public health policy planning and future research on these topics. Linking the Dental Health Register to other national data will generate unique possibilities for research not possible in many other settings. [12, 18, 30–32]

## Conclusion

For patients coded as having less than 32 remaining intact teeth but not being edentulous the reported number of remaining and intact teeth is to a very high degree correct. However, the correctness for those coded as edentulous or having 32 remaining intact teeth is low and varies substantially by age. The high validity of reported remaining and intact teeth in the Swedish Dental Health Register offers enormous potential for descriptive and analytical population-based dental health research.

## Data Availability

According to Swedish Law the data cannot be placed in a publicly available repository. Researchers can after ethical approval apply for data from the National Board of Health and Welfare, Stockholm, Sweden at www.socialstyrelsen.se. The Swedish national registers offer great potential for population-based register research. We recommend researchers unfamiliar to register-based research and researchers from outside of Sweden to collaborate with experienced researchers in Sweden, the authors (Dr Ljung) can facilitate such contacts.

## Abbreviations

CI: Confidence Interval
PPV: Positive Predictive Value
